# The effects of low tidal ventilation on lung strain correlate with pulmonary compliance

**DOI:** 10.1186/2197-425X-3-S1-A310

**Published:** 2015-10-01

**Authors:** J Xie, F Jin, C Pan, S Liu, L Liu, J Xu, Y Yang, H Qiu

**Affiliations:** Nanjing Zhongda Hospital, Southeast Unversity, Critical Care Medicine, Najing, China; Nanjing Zhongda Hospital, Southeast University, Nanjing, China

**Keywords:** Acute respiratory distress syndrome, mechanical ventilation, tidal volume, lung strain, ventilator induced lung injury

## Background

The effect of changing tidal volume on mortality of acute respiratory distress syndrome (ARDS) is determined by pulmonary compliance. We aimed to investigate the effects of different tidal volumes on lung strain in patients with ARDS.

## Methods

Nineteen patients were divided into high (C_high_ group) and low pulmonary compliance (C_low_ group) groups based on their pulmonary compliance values (0.6 ml/[cmH_2_O·kg]). End-expiratory lung volumes (EELV) with different tidal volumes (V_T_ at baseline and at 6, 8, 10 and 12 ml/kg predicted body weight [PBW]) were measured by nitrogen wash-in/washout. Lung strain was calculated as the ratio between tidal volume and EELV.

## Results

The mean baseline EELV and pulmonary compliance values were 1873 ml, 0.31 and 0.65 ml/(cmH_2_O·kg), respectively; all differences were statistically significant between the two groups. For all participants, a positive correlation was found between pulmonary compliance and EELV (r^2^ = 0.238, *p* = 0.034). When the tidal volume increased gradually from 6 ml/kg to 12 ml/kg also increased gradually, from 0.31 ± 0.27 to 0.52 ± 0.46, which represented a positive correlation (r^2^ = 0.956, *p* < 0.01). In the C_high_ group, there were no significant differences in strain between the tidal volume ventilation of 6 ml/kg.PBW and the volumes of 8 and 10 ml/kg.PBW. However, strain increased significantly with the tidal volume of 12 ml/kg.PBW. The mean lung strain was < 0.27 with ventilation at tidal volumes of 6 and 8 ml/kg.PBW, whereas it was > 0.27 with volumes of 10 and 12 ml/kg.PBW. In contrast, in the C_low_ group, there were no significant differences in strain between the different tidal volumes. However, mean lung strain was ≥ 0.27 for each tidal volume. In addition, 60% of patients' lung strain measured ≥ 0.27 even with tidal volume ventilation of 6 ml/kg.PBW.

## Conclusions

Pulmonary compliance affected the effect of tidal volume on strain in ARDS patients, which suggests that setting individual tidal volumes based on pulmonary compliance and strain may be more rational.

## Trial Registration

Clinicaltrials.gov identifier NCT01864668

